# Suggestions as to the Method of Using Narcotics in Nervous Diseases

**Published:** 1855-09

**Authors:** John R. Allen

**Affiliations:** Prof. of Obstetrics and Diseases of Women and Children in Iowa Medical Department


### SUGGESTIONS AS TO THE METHOD OF USING NARCOTICS IN NERVOUS DISEASES.

(CONCLUDED.)

BY JOHN R. ALLEN, M. D.,

Prof. of Obstetrics and Diseases of Women and Children in Iowa Medical Department.

Now any one will see from the physiological action of narcotics, that they will have a tendency to counteract these symptoms, and where there are no accompanying contra-indications, most practitioners would at once, and very correctly, prescribe them. But after all who uses them for any other purpose than to palliate the varied neurosis, whether attended with inflammation or not? How many allow them a curative agency?

Since the first part of this article was published, I have met in the New Orleans Medical and Surgical Journal, an article from the pen of a very intelligent writer, Dr. M. M. Dowler, in which he has quoted from Prof. Paine, of University of New York, remarks which we had also selected, illustrative of the views generally entertained as to the nature and curative value of narcotics. We still incorporate them, feeling much gratified to find our own views coinciding with those of Dr. Dowler, as to the true merits of this class of remedies.

We refer our readers with great satisfaction to the article referred to by Dr. D., as suggestive of valuable thoughts as to the use of these agencies in phlegmasial diseases:

"On the contrary," says Professor Paine, "I have endeavored to show in the various parts of this work, that narcotics are but little more than humble auxilliaries to more important remedies, and then only in a comparatively small number of cases of disease; or they are mere palliatives, giving a temporary ease by blunting sensibility where death is probably inevitable, and thus easing the sufferer out of existence."

"That narcotics are extremely deficient in curative virtues, should be sufficiently apparent from what has already been said of the uses to which they are constantly applied. But even these intentions can rarely be fulfilled by narcotics, where much disease is present. We must resort to the class of antiphlogistics for our curative means; and, if the narcotics be summoned to their aid, it should be done with the greatest caution, or they may prove fatally morbific. We may exhibit opium, &c., for the relief of mere spasm of the stomach, or to procure rest, &c., where no important acute disease is present. But he who should employ them to assuage the pain of pleuritis, enteritis, or any other active form of inflammation, and, in a general sense, of chronic forms, would either most seriously aggravate the disease, or destroy the patient.

"Whenever there is any affection of the head, or any tendency to cerebral disease, so great is the liability of narcotics to induce congestion of the brain, that they are totally inadmissible where that organ is increased in its susceptibilities. And let us consider their never-failing effect in their ordinary doses, of so injuriously modifying the action of the glandular organs, that the secretions of the whole, especially of that most important organ the liver, are more or less diminished; whereby nature is obstructed in one of her greatest processes natural and curative; morbific influences are reflected upon all diseased parts, and upon the whole organism. Should there be set up in the skin a perspirable action, it is not of a salubrious nature; and here again we see the evils that arise from regarding the product, and not the nature of the action upon which it depends." * * *

"The most extensively useful effect of narcotics is that of procuring sleep." * * *

"In serous inflammation of the bowels, on the other hand, they [opiates] are entirely inadmissible. * ** It is in all such instances a subordinate agent. * * * The pain of mucous and serous inflammation of the intestines may be exactly the same, and opiates curative in the former, but certainly fatal in the latter."—*Institutes of Medicine*, pp. 584–6.

"In respect to the agents now before us, (narcotics) there is a yet smaller class who are equally unhappy in their estimate of their virtues; and while the *stimulating* school exhaust the energies of nature by adding to the intensity of the disease in their peculiar way, the *narcotizing* school do the same mischief by a similar neglect of the pathology of disease; and what, in either case, should be attacked by the lancet, cathartics, antiphlogistic alteratives, &c., is roused into greater immediate violence by tonics and stimulants, or indirectly by other morbific influences which appertain to the narcotics."—*Ibid.* p. 584.

What are the effects of a medium dose of these remedies? primarily, but transiently stimulant, but ultimately anodyne and in full doses hyynotic—In other words, their effect is, to allay nervous excitement, to control the nervous force.

This effect follows soon after their administration, and is an effect which may be maintained for any length of time by a repetition of doses in quantities commensurate with the increasing tolerance of the economy. Where the object is to alleviate acute pain, or relax spasm, or quiet convulsions, we resort to decided and repeated doses and obtain the immediate repose desired; and there can be no estimate of the value of these agents here. But in cases of habitual neuralgia or spasm, or cases affecting the mental faculties, and the object is not to give immediate relief, for we can not hope to do this, but to remove a persisting cause from which the symptoms arise, another mode of procedure is desirable.

It is here that I think we are at fault, usually. We lay aside as but palliatives a class of remedies which should be regarded as curative. We forget that the suspension of the pain, or temporary control of the nervous force, may by being maintained for a long time, enable the affected part to return to a normal state, which would not have been gained in an equal contest with the irritation and perturbation, unaided by the soothing influences of the remedy.

I do not pretend to say how the change is brought about, whether it results from an alteration in the nutrition of the nerve fibre, or by a suspension of nerve force induced by the medium by which irritation is held in obeyance until the nervous equilibrium is established, or otherwise. I only know that it is often brought about, and in a most gratifying manner. I have seen such results, it is true, mostly in cases involving that part of the nervous contact appropriated to mental and moral manifestation, and have not had sufficiently extensive experience in other forms of nervous disorder to utter an opinion of much value. Regarding, as I have remarked, mental disorder and spasm to the brain and moter nerves, what pain is to those of sensation, I can see no reason why a similar course of treatment may not be as often successful in the one as the other,

The mode of application which I would suggest, is not that, as I

have remarked, looking to immediate results, but to a permanent, a prolonged narcotic effect—an *actual course*, if you please, to be persevered in as we do such plans of medication, with iron, mercury, arsenic, &c., when we desire to eradicate some inveterate morbid process, which we can not reach by any direct and decisive attack.

The plan I propose is to begin in such cases with whatever particular remedy we may select of this class, in a medium dose, and repeat as often and increase as much, as may be needful to produce and maintain such a state of ease or quietude as may be required. Quantity is not to be estimated, effect is to be looked for and aimed at.

Suppose I should say I have given Laudanum, Tinct. Hyasm. &c., &c., in ten drachm doses every four hours, for months at a time, and with the best results, in insanity and some forms of epilepsy! Surprising as this may appear, I will add that I have given *more than twice that dose*, and in the same way, with the greatest benefit. Nor am I alone in this, others have done so. I could cite case after case, thus treated successfully.

This course as will be perceived, was not intended for merely allaying the urgent calls of the case. If this happened well and good, but not being the expected result, the remedies were continued until the danger of recurrence was over. The nerve force is not only to be restrained but held in check, until we find, upon withdrawing gradually, as we had increased by degrees, the remedies, that the symptoms do not return.

They may be administered internally and applied externally, as the location and nature of the particular case may demand, but the principle of the treatment recommended is, to strive for a permanent effect to be wrought upon the nervous force or tissue, by a persistent narcotic impression, so prolonged, that it shall break up the morbid process, or destroy the morbid habitude of the nervous force.

I should transcend the intended measure of this article by going into detail on the application of narcotics in neuroses, and fearing in the hurry of preparation of this paper, I may not have made myself understood, I submit the following propositions:

1st. There is a morbid state of the nerve force, manifested by delirium, spasm, pain or paralysis, independent of any appreciable structural lesion.

2nd. Narcotics have a controlling influence over the nerve force, exercised in proportion to the dose administered, as to degree, and in proportion to the repetition and augmentation of the amount administered as to persistence of effect.

3d. In disorders of this nerve force of a persistent or habitual character, it is essential to the success of a narcotic treatment, that a prolonged impression be maintained upon the nervous functions in order to remove them.

4th. The method of administration should be by commencing with medium doses, and then a gradual increase to meet the increasing tolerance of the system for the remedies; the *effect* not the *quantity* to be the measure of their prescription.

It is now too late to question the efficacy of narcotics in incipient inflammations wherever seated, and in very active inflammation in particular localities, however high the authority for so doing.

No one doubts their applicability in painful diseases, acute or chronic, as palliatives at least. If we can thus momentarily relieve, why may we not prolong the impression which can be thus temporarily made, so that the *painful habitude* so to call it, may be interrupted and the normal powers overcome the tendency to neuralgia, while thus checked by medicines?

To this latter question we ask the attention of the profession.—The suggestions we have made are directed to this point, and we do not fear the practical results, whatever may be thought of the *modus operandi* which we have supposed to obtain.

At some future time we hope to be able to pursue more at length and more at leisure, this very interesting topic. For the present we must content ourselves with these hasty thoughts, trusting hereafter to illustrate the opinion inculcated by the results of their application.

